# Hydrogen Embrittlement at Cleavage Planes and Grain Boundaries in Bcc Iron—Revisiting the First-Principles Cohesive Zone Model

**DOI:** 10.3390/ma13245785

**Published:** 2020-12-18

**Authors:** Abril Azócar Guzmán, Jeongwook Jeon, Alexander Hartmaier, Rebecca Janisch

**Affiliations:** Interdisciplinary Centre for Advanced Materials Simulation (ICAMS), Ruhr-Universität Bochum, 44801 Bochum, Germany; jeongwook.jeon@rub.de (J.J.); alexander.hartmaier@rub.de (A.H.)

**Keywords:** density functional theory, hydrogen embrittlement, hydrogen enhanced decohesion, grain boundary segregation

## Abstract

Hydrogen embrittlement, which severely affects structural materials such as steel, comprises several mechanisms at the atomic level. One of them is hydrogen enhanced decohesion (HEDE), the phenomenon of H accumulation between cleavage planes, where it reduces the interplanar cohesion. Grain boundaries are expected to play a significant role for HEDE, since they act as trapping sites for hydrogen. To elucidate this mechanism, we present the results of first-principles studies of the H effect on the cohesive strength of α-Fe single crystal (001) and (111) cleavage planes, as well as on the Σ5(310)[001] and Σ3(112)[1$\bar{1}$0] symmetrical tilt grain boundaries. The calculated results show that, within the studied range of concentrations, the single crystal cleavage planes are much more sensitive to a change in H concentration than the grain boundaries. Since there are two main types of procedures to perform *ab initio* tensile tests, different in whether or not to allow the relaxation of atomic positions, which can affect the quantitative and qualitative results, these methods are revisited to determine their effect on the predicted cohesive strength of segregated interfaces.

## 1. Introduction

Hydrogen embrittlement (HE) is one of the main challenges in modern materials science, notably affecting structural materials, such as iron and steel. Different HE mechanisms are known, but their details and their interactions are not yet fully understood. An important mechanism is hydrogen enhanced decohesion (HEDE), which has been observed in ferritic microstructures [1]. Decohesion is defined as a sequential tensile separation of atoms ahead of a crack tip when a critical crack-tip-opening displacement (in the order of half the interatomic spacing) is reached. In the HEDE process, hydrogen accumulates between crystallographic planes, for example, due to strain fields, or by segregation to interfaces such as grain boundaries (GBs). There, it is assumed to reduce the interatomic bond strength, thus reducing the cohesive strength between the planes, and causing brittle fracture. Since GBs can act as H trapping sites and decrease the amount of mobile H atoms in the system [2], HEDE is expected to be most effective at these defects. Experimental observation of the process itself is problematic due to the high diffusivity of H and a fast failure process. Thus, atomistic studies are the method of choice to enhance our understanding of HEDE. So far, however, they have not produced a clear picture of intra- and intergranular HEDE.

In a study of HE with tight binding (TB) calculations using a cohesive zone model, Katzarov and Paxton [3] demonstrated that the cohesive strength across (111) cleavage planes in α-Fe is considerably reduced by the segregation of H to the cohesive zone, predicting enhanced intragranular HEDE in bcc Fe. They observed a reduction in cohesive strength from 33 GPa to 22 GPa with increasing H concentration, and this reduction mostly remains unaffected by a temperature change from 300 to 200 K and bulk hydrogen concentration in the range of 0.1 to 10 appm. In contrast, studies on GBs have not reported such a decrease in strength due to the dissolution of H. Tahir et al. [4] studied the Σ5(310)[001] symmetrical tilt grain boundary (STGB) in bcc with segregated C and H atoms using density functional theory (DFT). While they observed a decrease in the work of separation when H was placed in the GB plane, the same decrease was not observed in the theoretical strength. It was only reduced by 6% by a full monolayer of H. Similarly, Momida et al. [5] did not report a substantial reduction of the strength due to H solution at the Σ3(112)[1$\bar{1}$0] STGB in bcc Fe, only a 4% decline for a full monolayer. The study of the Σ5 STGB utilized rigid grain shifts, while for the Σ3 STGB tensile tests with relaxed atomic positions were employed. Contrary to the expectations of the authors, HE was not observed by the simple presence of H in the studied STGBs, and a possible embrittlement was attributed to interactions of H with other point defects. H can reduce the amount of sites available at the GB plane to be occupied by C, thus suppressing the important cohesion enhancing effect of the latter [4]; or it can interact with vacancies at the GB, thereby forming defect complexes and causing brittle fracture in steel [5].

Different aspects of the effect of H on the mechanical properties and GB cohesion have been investigated to explain the possible effects of H on cohesion in Fe. Psiachos et al. [6] found that the decrease of the elastic moduli with the presence of H in bcc Fe could be attributed to a detrimental volumetric effect. In the grain boundaries, the thermodynamic theory of Rice and Wang defines the effect of a solute on the GB cohesion through the segregation energy, furthermore, the effect can be separated into chemical and mechanical contributions [7,8]. The contributions reported in the literature for Fe GBs are somewhat ambiguous. On the Σ3(111) GB in bcc Fe, Yuasa et al. [9] found the charge density reduces with increasing strain when H is in the GB and Tian et al. [10] found the embrittling effect of H is due to the chemical contribution with no mechanical effect. While, on the Σ5(310) GB the embrittling effect is attributed to the mechanical contribution and no chemical effect is observed [4]. Finally, Zhou et al. [11] studied H trapping in fcc metals at the Σ5(210) GB and derived model based on the volumetric deformation of polyhedral structural units at H segregated GBs, meaning that the H adsorption at the GB depends on the excess volume and the coordination of around H.

This review shows, that the different predictions for H in Fe bulk and at GBs could indeed be due to a physical effect, i.e., that different local atomistic structures result in a lower or higher sensitivity to a reduction in bond strength by H. However, since the different studies differ in the chosen description of the interatomic interaction (within DFT or using a TB model) as well as in the relaxation during the tensile test procedure, this can not be concluded yet. In the study at hand, different structures are investigated with different tensile test methods to close this gap.

The study of inter- and intragranular fracture phenomena is complex and presents modelling challenges that are addressed on several length scales. Continuum descriptions use a cohesive zone model [12], which provides a traction-separation curve that characterizes the stress associated with the separation of two adjacent crystallographic planes. The characterization of the bond breaking that takes place in the cohesive zone can be improved with information from first-principles simulations. The *ab initio* tensile test has been vastly used to describe decohesion across cleavage planes as well as at GBs in pure and segregated systems [13]. However, the procedure followed to carry out the tests has been shown to affect the results, including a size effect when the relaxation of the atomic positions is allowed, due to the release of elastic energy as described by Nguyen and Ortiz [14]. Hence, different approaches have been taken to treat these effects and guarantee the comparability of different studies. One of such approaches was proposed by Van der Ven and Ceder [15,16], who linked first-principles calculations (with and without atomic relaxations) to the cohesive zone model through thermodynamic excess quantities. In such a first-principles cohesive zone model, only the bond breaking at the tip of the crack is included, which enables a unique scaling of the traction-separation law to the relevant length scale [17]. Impurity segregation to the crack complicates the picture of decohesion further. The consequences of segregation for decohering interfaces were established from a thermodynamic point of view by Rice, Hirth and Wang [7,18]. They defined two limiting cases: separation under constant chemical potential, for which the diffusion of the impurity to the interface is faster than the separation itself; and separation under constant solute concentration, where diffusion is slower and some segregation sites remain empty during the separation. Both conditions can be realized via the coupling of the cohesive zone to the corresponding thermodynamic ensemble [15,19].

The objective of the present work is to elucidate the HEDE mechanism in Fe and the discussed discrepancies between the bulk cleavage and GB planes. Furthermore, it is essential to determine if different methods of the tensile test result in the same or different predictions about the effect of H on Fe cohesion. To this end, tensile tests are performed on α-Fe with H impurities, using DFT [20,21] calculations to investigate the decohesion across (001) and (111) planes and of Σ5(310)[001] and Σ3(112)[1$\bar{1}$0] STGBs. Traction curves are obtained under the first-principles cohesive zone model when atomic positions are relaxed, as well as by using rigid tensile tests. This study focuses on the constant solute composition limit to model the decohesion of the segregated interfaces.

This paper is organized as follows: Section 2 describes the simulation details and the excess energy model used. The results are shown in Section 3, first the pure α-Fe, then α-Fe with H impurities and finally, the extension for the Fe GB with segregated H. Next, the discussion of the results in Section 4 and conclusions are drawn in the final section.

## 2. Methodology

### 2.1. First-Principles Cohesive Zone Model

A cohesive zone model [12] provides the coupling of a decohering region to a homogeneous part of the material. Traditionally, these models are used for all varieties of damage and fracture, and the details of the failure process are reflected in the shape of the traction-separation law, which describes the restoring stress perpendicular to the surface upon an opening of the cohesive zone. Recently, atomic-scale cohesive zone models have been promoted [15,19], in which the traction- separation only describes the breaking of the interatomic bonds [17]. All other dissipative processes have to be described outside the cohesive zone. This ensures the transferability of the traction-separation law to different loading scenarios, and provides the possibility to feed *ab initio* DFT data directly into a continuum model. On the atomic scale the decohesion can be studied through the so-called *ab initio* tensile test, where a certain displacement is introduced in the supercell along the loading axis. The energy, *E*, is calculated at different displacements, Δ, and the stress, *σ*, is obtained from

(1) $$\sigma =\frac{1}{A}\frac{\partial E}{\partial \Delta }.$$

#### 2.1.1. Effect of Relaxation of Atomic Positions

The procedure to introduce said displacement in the supercell or the relaxation scheme of the atomic coordinates and axes can have an impact on the final results of such calculations. In the present work the tensile tests were carried out following two procedures:

(i) rigid grain shifts (RGS): displacement is concentrated between two blocks of atoms or “grains” defining the cleavage plane (due to periodic boundary conditions, two cleavage planes or cohesive zones are created in a GB supercell). The energy is obtained after a rigid calculation with fixed atomic coordinates.
(ii) rigid grain shifts with subsequent relaxation (RGSrel): using the same procedure as in previous case but allowing the relaxation of the atomic positions at each displacement. This relaxation releases elastic energy.

In all tensile tests performed in this work the transverse supercell dimensions are kept fixed, suppressing Poisson contractions. From this uniaxial strain loading one can derive a traction-separation law for continuum fracture simulations under mode I loding and plane strain conditions. In this case, a triaxial stress state is expected at the crack tip, which corresponds well to the stress occuring in the *ab initio* tensile test without lateral contraction [17,22,23].

**Figure 1:**
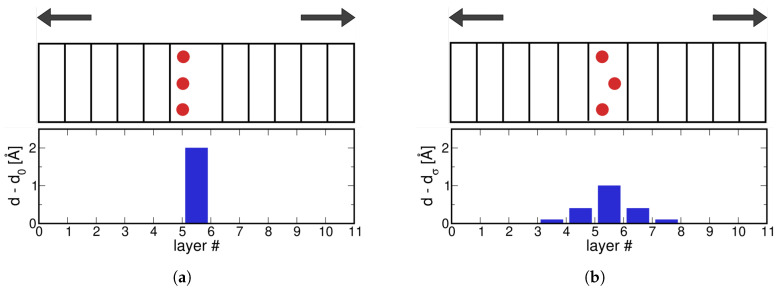
Schematic representation of the *ab initio* tensile test with a defect (top) and the distribution of the deviation in interplanar distance among the crystallographic layers (bottom), in the case of (**a**) rigid grain shifts (RGS) and (**b**) RGSrel. d_0_ (d_*σ*_) is the equilibrium interplanar spacing at zero (finite) stress.

The main difference between cases RGS and RGSrel is in the displacement, Δ, in Equation (1). The total displacement of the supercell is an ill-defined quantity in case the strain can redistribute during a relaxation of the atomic positions. Figure 1 presents a final picture of the atomic layers in both cases. Red atoms indicate the presence of impurity atoms in the decohering plane, but this schematic applies to all supercells with a defect (e.g., segregating atoms or an interface). Also in the schematic is the interplanar distance difference per layer, where *d* is the interplanar distance or interlayer spacing in the loading direction at a certain stress and $d_{0}$ is the interplanar distance at equilibrium or zero stress. In the RGS case, the displacement is introduced between two layers and all other layers have an interlayer distance equal to $d_{0}$. In the case with relaxation, RGSrel, the displacement distributes among several layers, to an extent which depends on the type of defect. When a first-principles cohesive zone model is used, the cohesive zone refers to the bond breaking at the tip of the crack. In the rigid tensile test, the cohesive zone is retained between the two decohering planes, while in a relaxed tensile test the cohesive zone extends in several layers.

#### 2.1.2. Universal Binding Energy Relationship (UBER)

To find an analytic expression for the energy in the case of a rigid tensile test is rather straightforward, due to the formulation of Rose et al. [24] where the binding energy of metals, *e_b_*, follows a universal behavior known as the UBER fit:

(2) $$e_{b}\left(\Delta \right)=\left|e_{b}^{0}\right|\left(-1-\frac{\Delta }{\lambda }\right)\exp\left(-\frac{\Delta }{\lambda }\right),$$

where $|e_{b}^{0}|$ is the binding energy at equilibrium, equal to the negative of the work of separation ($W_{sep}$), since the surface energy, 2*γ*, is subtracted from all energies. The displacement, Δ, is scaled by the characteristic length *λ*, a value that depends on the curvature at the minimum, Δ^0^:

(3) $$\lambda =\sqrt{\frac{|e_{b}^{0}|}{e_{b}^{\prime \prime }\left(\Delta^{0}\right)}},$$

where $e_{b}^{\prime \prime }\left(\Delta^{0}\right)$ is obtained through the second derivative of a polynomial fit around the minimum. The UBER fit can also be used for the relaxed energies of GB supercells, however, in this case *λ* becomes a fitting parameter, no longer derived from the curvature at the minimum.

#### 2.1.3. Excess Quantities

Van der Ven and Ceder [16] derived a cohesive zone model which accounts for atomic relaxations based on excess quantities obtained from the difference between energies obtained via rigid grain shifts and a homogeneous elongation of the single crystal. In the case of the latter, no cleavage plane is defined but strain is introduced by scaling the length of the supercell perpendicular to the tensile axis. In such a homogeneously elongated crystal (HEC) the relaxed, equal distribution of the strain among the crystallographic planes is preserved naturally. The excess energy is calculated as

(4) $$e_{ex}\left(\sigma \right)=\frac{E_{RGSrel}^{tot}\left(\sigma \right)}{A}-\left(n_{p}-1\right)\frac{E_{HEC}\left(\sigma \right)}{A}$$

where $E_{RGSrel}^{tot}$ is the total energy of the supercell from the RGS with relaxation and $E_{HEC}$ the total energy *per atomic plane* from the homogeneously elongated single crystal supercell. The variable *n_p_* denotes the number of atomic planes perpendicular to the tensile axis and *A*, the area projected onto the cohesive zone. Both energies are at the same stress, *σ*, further details on this aspect are shown in Section 2.1.4. Similarly, the excess length is defined as

(5) $$l_{ex}\left(\sigma \right)=L_{RGSrel}^{tot}\left(\sigma \right)-\left(n_{p}-1\right)L_{HEC}\left(\sigma \right),$$

where $L_{RGSrel}^{tot}$ is the total length of the supercell in the elongation direction of the RGSrel test and $L_{HEC}$, the length between two adjacent planes in the HEC case. Finally, the separation or opening of the cohesive zone can be expressed as

(6) $$\delta =l_{ex}-d_{0},$$

with *d*_0_ the equilibrium interplanar distance at zero stress. The excess energy, $e_{b}\left(\delta \right)$, can be fitted using the UBER fit from Equation (2) w.r.t. the opening of the cohesive zone instead of the displacement as the case for the rigid tensile tests.

While the calculation of the excess quantities removes any size-dependencies of the relaxed energies, it leads to the problem, that not the whole excess energy vs. opening curve can be accessed. During tensile decohesion, two stable states exist, a bulk like state, in which the opening strain is re-distributed among the crystallographic planes, and the decohered surface slabs. These two states are separated by an energy barrier [13,25], thus, there is no continuous transition from one to the other. Instead, the lattice planes relax back to the bulk like state below a critical separation; above it, they relax to the surface state. As shown in Reference [13], the resulting region of inaccessible openings becomes larger with increasing system size. Choosing the cell size thus becomes a compromise—the cell should have enough layers to avoid interaction between the defects but a higher size results in more of the curve being inaccessible.

#### 2.1.4. Stress and Interplanar Distance

During a first-principles tensile test, two systems are at the same stress, if the interplanar distance in the loading direction, *d*, in both systems, is equal. In the case of RGSrel procedures, the interplanar distance is taken at the center of the “grain”; in other words, sufficiently far from the cohesive zone or from the effect of any segregated impurities or GB, if present. Furthermore, the stress as a function of interplanar distance can be obtained from the energies of a homogeneously elongated crystal.

**Figure 2:**
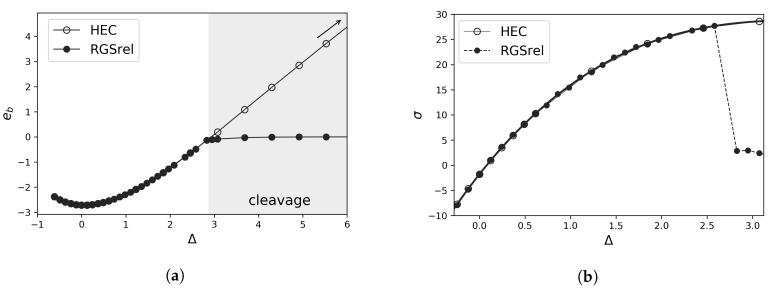
(**a**) Energy during tensile tests using a homogeneously elongated crystal (HEC) or rigid grain shifts with relaxation (RGSrel). The gray area refers to the decohered state in the RGSrel case. (**b**) Traction-separation curve derived from the polynomial fit of the HEC energies and the interplanar distance on the RGSrel case.

A schematic representation of the energies obtained from such calculations, in which either a single crystal is homogeneously elongated, HEC, or the cleavage plane is predefined, RGSrel, are shown in Figure 2a. There are two states of deformation: for small displacements, the crack in the RGSrel case heals back to a homogeneously elongated state, and both HEC and RGSrel procedures result in the same interplanar distance. For higher displacements (gray area), the RGSrel results in cleavage or two separated grains, in which *d* relaxes back to the equilibrium value at zero stress, while in case of a HEC it continues to increase until a point of inflection appears in the energy as the structure becomes unstable and separates into *n_p_* individual planes.

The HEC energy curve can be fitted with a polynomial function and the stress calculated from Equation (1) (HEC curve in Figure 2b). Finally, this stress is assigned to the RGSrel data when it has the same interplanar distance (RGS rel data in Figure 2b). Note that the maximum stress from a RGSrel calculation can not be interpreted as the cohesive strength, since the elastic energy which is released during the decohesion depends on the supercell size [25]. Hence, the excess length and energy have to be considered to get rid of any size-dependency in the cohesive strength value.

### 2.2. Simulation Cell Construction

**Figure 3:**
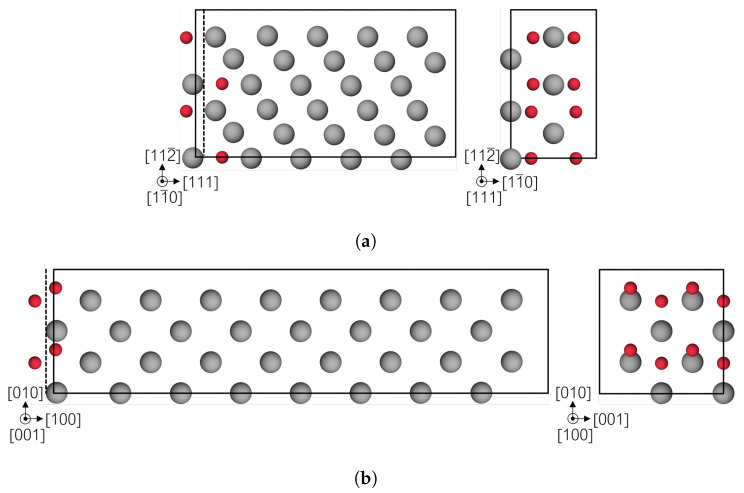
Atomic structure of bcc Fe (gray atoms) with segregated H (red atoms), the cleavage plane for the decohesion study is represented by the dotted line. (**a**) (111) plane and (**b**) (001) plane.

To investigate the H effect on the decohesion of bcc Fe, two different single crystal supercells were created to obtain the (001) and (111) cleavage planes. The (111) supercell is shown in Figure 3a. The lattice vectors of the cell are $2.5a_{0}\left[111\right]\times a_{0}\left[11\bar{2}\right]\times a_{0}\left[1\bar{1}0\right]$ with a total of 30 Fe atoms. Since hydrogen occupies tetrahedral sites in Fe bulk, there are 12 possible interstitial sites between two (111) crystal planes. To probe the optimal configurations for cells with H, a structure with full occupation of the 12 sites was relaxed and it was observed that H atoms separate in 3 different layers, of 4 atoms each, separated 0.8 Å. Three different concentrations for H were selected filling up to 4 atoms per layer: Fe_30_H_1_, Fe_30_H_4_ and Fe_30_H_8_, the last having two layers of H atoms as depicted in Figure 3a. Respectively, the areal concentrations are 0.04, 0.14, 0.29 atom/Å^2^, obtained from the number of H atoms per area of the cleavage plane. The lattice vectors for the (001) supercell are $8a_{0}\left[100\right]\times 2a_{0}\left[010\right]\times 2a_{0}\left[001\right]$ with 64 Fe atoms. The structure is shown in Figure 3b. Cells with for different concentrations of H were created: Fe_64_H_1_, Fe_64_H_2_, Fe_16_H_1_ and Fe_16_H_2_, that is in terms of areal concentration: 0.03, 0.06, 0.12 and 0.25 atom/Å^2^, respectively. The periodic boundary condition allows the two highest concentrations to be achieved with a smaller supercell, which was used to reduce computational costs. One face of the bcc unit cell contains 4 interstitial sites, which translates to 16 possible positions for the H atoms in the supercell. In the case of Fe_64_H_2_, this configurational space was probed and the structure with the lowest solution energy was selected. Similarly, the most favorable case in Fe_16_H_2_ was dividing the H atoms in two layers, as the highest concentration in the (111) supercell.

**Figure 4:**
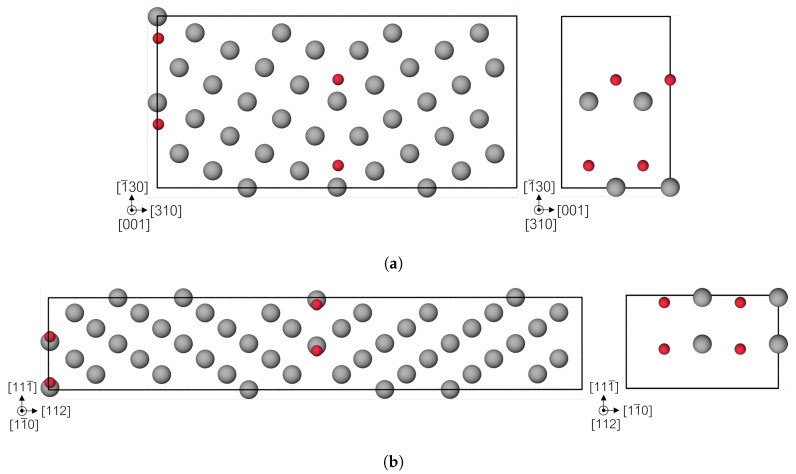
CSL models of the bcc Fe (gray) (**a**) Σ5 and (**b**) Σ3 symmetrical tilt grain boundaries (STGBs). On the right a view of the GB plane with the segregation sites for H atom (red).

For the comparison of the decohesion behavior in Fe bulk with grain boundaries, two symmetrical tilt GB supercells were constructed: Σ5(310)[001] and Σ3(112)[1$\bar{1}$0]. The former has a misorientation or tilt angle of 36.9° and the lattice vectors are: $3a_{0}\left[310\right]\times 1.5a_{0}\left[\bar{1}30\right]\times 2a_{0}\left[001\right]$. It contains 10 atomic layers between each GB plane. A visualization of the 80 Fe atom cell can be found in Figure 4a. The view of the GB plane (310) shows there are 4 possible segregation sites for the H atom, which are occupied in increasing steps of one atom at a time, creating the structures: Fe_80_H_2_, Fe_80_H_4_, Fe_80_H_6_ and Fe_40_H_4_ (for both the pure Fe and full occupation of H the [001] supercell vector is divided by 2, achieving the same structure with 40 Fe atoms due to periodic boundary conditions). The corresponding areal concentrations are: 0.02, 0.04, 0.06 and 0.08 atom/Å^2^. The other GB supercell is the Σ3 with a tilt angle of 70.53° and lattice vectors: $a_{0}\left[11\bar{1}\right]\times 4a_{0}\left[112\right]\times 2a_{0}\left[1\bar{1}0\right]$ with 12 atomic layers between the GB planes. Figure 4b shows the cell with a view of the segregation sites in the GB plane, which correspond to the octahedral sites. Only the case of full H occupation was taken, Fe_96_H_8_ equivalent to 0.05 atom/Å_2_. For the decohesion analysis, single crystal structures were created oriented in the same directions as the GB supercells, namely with (310) cleavage plane as reference for Σ5 and (112) for Σ3.

Note that this set-up for bulk and GB supercells leads to one cohesive zone in the bulk, but two in the GB cells.

The interface energy of a grain boundary can be calculated as,

(7) $$\gamma_{GB}=\frac{E_{tot}^{GB}-E_{tot}^{bulk}}{2A},$$

where $E_{tot}^{GB/bulk}$ refers to the total energy of a supercell with a GB or a bulk supercell with the same amount of Fe atoms, and the area of the GB plane, *A*, is taken twice due to the presence of two equivalent GB planes in the supercell.

The GB energies computed are in Table 1 and in good agreement with those reported in the literature [2,4,5,26,27,28,29,30,31,32]. The optimization of both GB supercells was carried out following the procedure described by Ochs et al. [33] and the excess length introduced in the cell perpendicular to the GB plane (difference between the length of cells with GB and single crystal) is also shown in Table 1. The Σ3 STGB has a lower energy and excess length as a result of the more closed-packed interface, as compared to the Σ5 STGB.

### 2.3. Computational Details

Density functional theory calculations were carried out as implemented in the Vienna Ab initio Simulation Package (VASP) [34,35]. The projector augmented-wave (PAW) method [36,37] describes the core-valence interaction, where the 3*p* electrons are explicitly treated as valence in Fe. The exchange- correlation energy was estimated with the generalised gradient approximation (GGA), using the Perdew-Burke-Ernzerhof (PBE) parametrization [38]. The calculations were performed in a spin polarized fashion to account for the magnetism in α-Fe. A plane-wave basis cut-off energy of 400 eV was used for all supercells, achieving a convergence of the solution energy of H within 2 meV/atom. Different *k*-point meshes of the Monkhorst-Pack type [39] were constructed for the different supercells. For the single crystal cases, the (111) cell has 2 × 4 × 6, the (001) 1 × 4 × 4 and the (001) reduced cell 1 × 8 × 8 *k*-points. The Σ5 STGB meshes are: 2 × 4 × 4 for 80-atom cell and 2 × 4 × 8 *k*-points for the 40-atom cell. For the Σ3 STGB, a 4 × 2 × 4 mesh was used. The first-order Methfessel-Paxton method [40] was used for Fermi surface smearing with a width of 0.1 eV. The equilibrium lattice constant $a_{0}$ for bcc Fe is 2.837 Å, in agreement with that of Hristova et al. [27]. The reference chemical potential of hydrogen is, $\mu_{H}$ = −3.385 eV, calculated in the H_2_ molecule.

## 3. Results

### 3.1. Pure Fe Bulk

**Figure 5:**
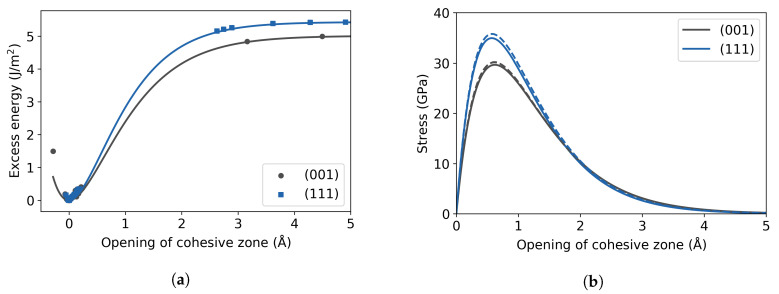
(**a**) Excess energy for different cleavage planes in pure bcc Fe. Solid line represents the UBER fit. (**b**) Traction curve derived from the excess energy(solid line) and from rigid energy (dotted line).

The first-principles tensile tests were carried out for the pure α-Fe cases, as described in Section 2.1, by introducing rigid separation in equidistant steps of 1% from −5% to +25%, and steps of 5% afterwards, with posterior relaxation of the atomic positions at each step. The excess energy from the decohesion in the (111) and (001) cleavage planes is illustrated in Figure 5a, including the corresponding universal binding curves. They exhibit the aforementioned fact, that the data of excess energy vs. opening of cohesive zone is separated into two sets, pertaining to before and after decohesion occurs, leaving a section of the curve without data (compare Section 2.1.3). The work of separation, $W_{sep}$, is 5.01 J/m^2^ for the cleavage of two adjacent (001) planes and 5.43 J/m^2^ for the (111) planes. This is comparable to the calculated DFT values by Spencer et al. [41], which are 4.69 J/m^2^ for (001) planes and 5.38 J/m^2^ for (111) planes. This suggests that the adhesion of two (111) surfaces is stronger than that of two of (001) and concurring with the experimental findings that (001) planes are the preferred cleavage planes in bcc Fe [42]. The value obtained for the (111) decohesion using magnetic TB calculations is 3.50 J/m^2^ [3].

The traction curves, obtained from Equation (1), are presented in Figure 5b. The stress is obtained from the excess energies (solid lines) and from the rigid energies (dotted lines) and both curves coincide for the two different planes, which was also observed by Van der Ven and Ceder [16]. This indicates that the main contribution to the energy comes from the two adjacent separating planes and not from neighboring atomic planes in pure bulk. The maximum of the stress curve is identified as the theoretical cohesive strength, found to be 30 GPa for the (001) plane and 35 GPa for the (111) plane. The latter value compares well to the one obtained with a TB model, 33 GPa [3] with the same calculation scheme. Note, however, that these cohesive strength values are significantly higher than the theoretical tensile strength of iron single crystals, which is obtained if a phase transition is permitted [43,44], that is, by performing a homogeneous elongation tensile test allowing Poisson contraction. Friák et al. found a critical stress of 12.7 GPa for the bcc → fcc transition when loading along the [001] direction, and 27.3 GPa for the bcc → sc transition when loading along [111]. This transition could be a mechanism to dissipate energy in front of a crack tip, instead of crack propagation by brittle cleavage.

### 3.2. Fe with H Impurities

Hydrogen enhanced decohesion in both of the chosen Fe cleavage planes was studied by varying the number of H atoms, $N_{H}$, in the cohesive zone. The H positions were assumed to be in tetrahedral interstitial sites, as in the undistorted single crystal and the areal concentrations obtained as described in Section 2.2. The excess energy from Equation (4) was modified to balance the energy of the added H atoms,

(8) $$e_{ex}^{H}\left(\sigma \right)=\frac{E_{RGSrel}^{Fe+H}\left(\sigma \right)}{A}-\left(n_{p}-1\right)\frac{E_{HEC}^{Fe}\left(\sigma \right)}{A}-N_{H}\mu_{H},$$

where $E_{RGSrel}^{Fe+H}$ are the total energies of the Fe supercells with segregated H atoms with relaxation of the atomic coordinates. $E_{HEC}^{Fe}$ is the total energy per plane of the homogeneously elongated pure Fe cell and $n_{p}$, the number of Fe layers.

**Figure 6:**
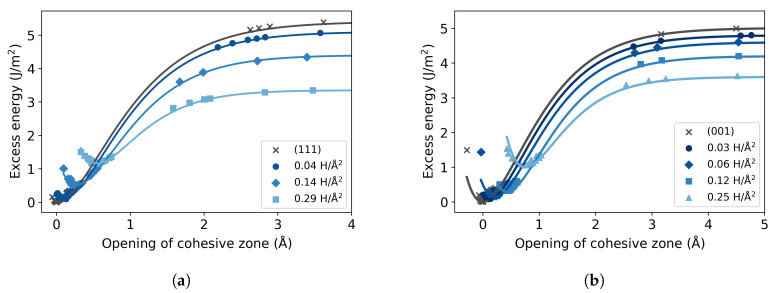
Excess energy at different areal concentrations of hydrogen, for the (**a**) (111) and (**b**) (001) cleavage planes. In each case the minimum excess energy of the pure bulk is subtracted.

The excess energy curves for the different H concentrations are illustrated in Figure 6. The origin of the plot is the energy of the pure single crystal without stress. Again, as mentioned in Section 2.1.3, the curves suffer from the fact that the region around the point of inflection is not directly accessible. Nevertheless, the fitted curves show a systematic trend. In the case of the (111) plane the work of separation is reduced to 2.09 J/m^2^ at the highest H concentration, while for the (001) plane the highest H areal concentration exhibits $W_{sep}=$ 2.50 J/m^2^. Since the opening of the cohesive zone is calculated with reference to the pure Fe HEC, following Equation (6), H introduces a shift of the minimum (i.e., a pre-opening of the cohesive zone), which increases with increasing concentration.

**Figure 7:**
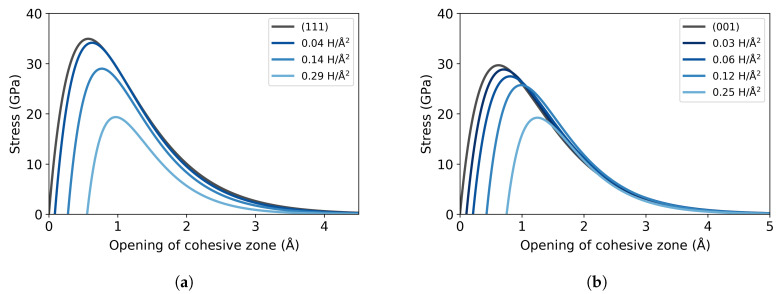
Traction-separation curves at different areal concentrations of hydrogen, for the (**a**) (111) and (**b**) (001) cleavage planes.

The traction curves are derived from the excess energy (Figure 7). As expected from the observed reduction in the work of separation, also the stress is reduced with increasing H concentration, for both studied cleavage planes. The cohesive strength of the (111) plane decreases by 45% from 35 GPa for pure Fe to 19 GPa at 0.29 atom/Å^2^. Similarly, the stress of the (001) plane is also reduced with the presence of H from 30 GPa to 19 GPa; this represents a 36% decline at 0.25 atom/Å^2^. The stress curves obtained for the (111) plane can be compared with the results of Katzarov and Paxton [3], who report that the stress decreases from 33 GPa for the Fe single crystal to 22 GPa at 0.29 atom/Å^2^, when there are 8 H atoms in the cohesive zone of the supercell transverse area. These values obtained with TB calculations indicate a 33% reduction for this H concentration and are in good agreement with our findings.

**Figure 8:**
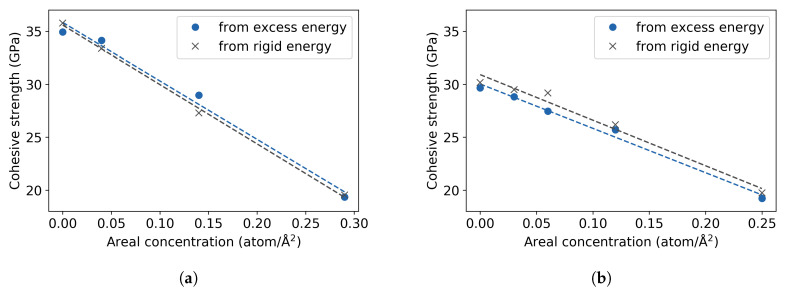
Theoretical cohesive strength vs. areal concentration of hydrogen from excess and rigid energies, for the (**a**) (111) and (**b**) (001) cleavage planes. Dotted line indicates linear fit of the data.

The theoretical cohesive strength is also calculated from the RGS tensile test with rigid energies and the results, as well as those from the excess energy, are shown in Figure 8. The calculated cohesive strengths from rigid energies for the (111) plane are essentially the same as the values obtained from the excess energies, a linear fit of both data sets coincides with negligible deviations. Similarly, the same decreasing trend is observed between both methods in the (001) plane.

### 3.3. Fe Grain Boundaries with H Segregation

**Figure 9:**
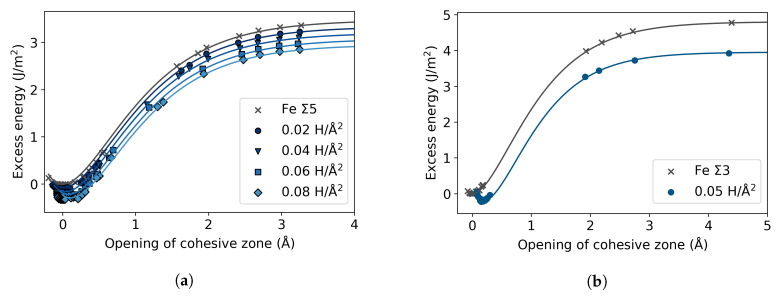
Excess energy at different areal concentrations of hydrogen, for the (**a**) Σ5(310)[001] and (**b**) Σ3(112)[1$\bar{1}$0] STGBs. In each case the minimum excess energy of the pure GB is subtracted.

To investigate the effect of H segregation on grain boundary cohesion, and hence on intergranular fracture, tensile tests following the RGS and RGSrel procedures were performed, in which the GB plane and the layer next to it were defined as separating planes. Accordingly, the separation in the GB supercells occurs in two cohesive zones. Apart from this, the same formalism previously described is applied for the calculation of the excess energy and length with a GB present in the system. The excess energy is obtained by subtracting from the total energies of the pure or segregated GBs ($n_{p}-1$) times the energy per plane of a homogeneously elongated Fe single crystal and dividing by two. In the excess energy curves shown in Figure 9, the opening of the cohesive zone for the Σ5 or Σ3 STGB is not zero, as this would be the case for pure Fe bulk. This shift is the excess length induced by the GB, $\Delta L^{GB}$, and it shifts further with the presence of H atoms. The excess length of the cohesive zone at the GB, $l_{ex}^{GB}$, is

(9) $$l_{ex}^{GB}\left(\sigma \right)=\frac{1}{2}\left[L_{RGSrel}^{GB}\left(\sigma \right)-\left(n_{p}-1\right)L_{HEC}^{bulk}\left(\sigma \right)\right]-\Delta L^{GB}.$$

In the case of Σ5, the effect of the H atoms in the cohesive zone is negligible with respect to the pure GB showing a shift of 0.03 Å at the maximum concentration, unlike the Σ3 case, where the GB with H increases to 0.19 Å. In a similar manner, the effect of H in the work of separation of the Σ5 STGB is slim, reducing from 3.36 J/m^2^ to 3.00 J/m^2^; while there is a more pronounced effect from the H in the Σ3 STGB decreasing from 4.79 J/m^2^ to 4.08 J/m^2^. The values are consistent with the previously reported results in the literature for these GBs [4,5,31,32]. Contrary to the bulk cleavage planes (Figure 6), the excess energy is reduced with the addition of H in the grain boundary. This is in agreement with the GB being attractive to the H atoms, that is, H having negative segregation energies at the GB [4,32].

**Figure 10:**
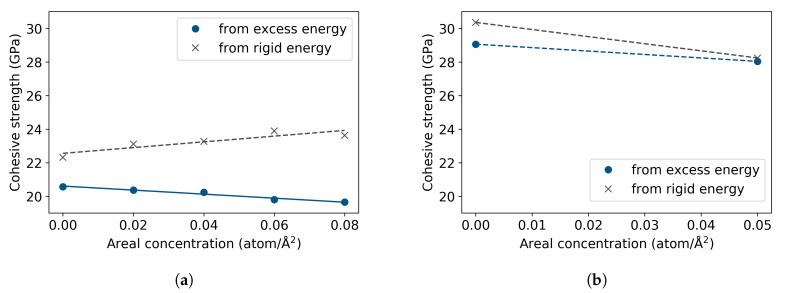
Theoretical cohesive strength vs. areal concentration of hydrogen from excess and rigid energies, for the (**a**) Σ5(310)[001] and (**b**) Σ3(112)[1$\bar{1}$0] STGBs. Dotted line indicates linear fit of the data.

From the traction-separation curves, the theoretical cohesive strengths were obtained and are shown in Figure 10. The results of the decohesion in Σ5 determined from the relaxed energies show a 3% reduction of the strength; contrarily, the rigid case indicates a 6% increase. The strength of the Σ3 STGB exhibits the same trend from both excess and rigid energies, decreasing from a 30 GPa rigid strength and 29 GPa relaxed strength to 28 GPa in the presence of H, in both cases.

## 4. Discussion

**Figure 11:**
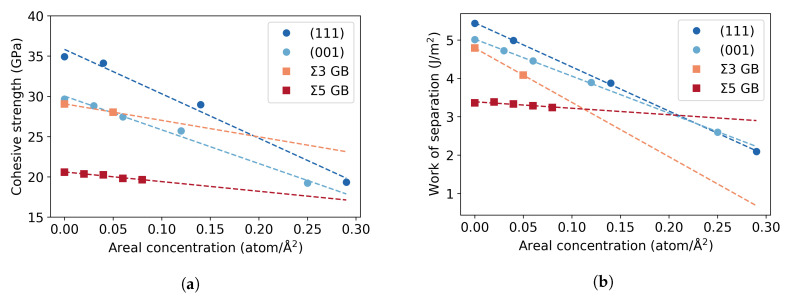
(**a**) Theoretical cohesive strength and (**b**) Work of separation vs. areal concentration of hydrogen extrapolated linearly to 0.29 atom/Å^2^.

The findings of this study show a decrease of the cohesive strength across (111) planes of 45% at a hydrogen coverage of 0.29 atom/Å^2^ and a reduction of 36% at 0.25 atom/Å^2^ across (001) planes (Figure 11a). A linear extrapolation to 0.29 atom/Å^2^ predicts a reduction of the cohesive strength across the (001) planes of 40%, meaning the reduction of strength by the presence of H is similar across the (111) and (001) planes. Such a significant decrease is not obtained in the grain boundary structures within the studied range of concentrations. The limit of this range is given by an occupation of all available low-energy segregation sites at the GB plane by one H atom, which in total also corresponds to one H monolayer. The cohesive strength of the Σ5 is reduced by 3% at a coverage of 0.08 atom/Å^2^ and the one of the Σ3 STGB by 6% at 0.05 atom/Å^2^. These concentrations are rather low, considering that H is supposed to accumulate at the GB. If the strength is extrapolated to 0.29 atom/Å^2^, as done for the single crystals, the reduction of the theoretical strength would be 12% for the Σ5 STGB and 21% for the Σ3 STGB (in Figure 11). Figure 11b presents also the work of separation, which completes the information necessary to define traction-separation laws. In this case, the trend for the Σ3 STGB is more similar to the bulk cleavage planes, which means that the reduction of the (112) surface energy with H is more pronounced than that of the (310) plane. The change in cohesive energy can formally be split into a chemical and mechanical contribution, see details in Reference [8]. It can be seen in Table 2 that the effect of H on cohesive energy is dominated rather by the chemical contribution than the mechanical one. Within the simple linear approximation and the studied concentration ranges, indeed no significant detrimental effect of H segregation on the decohesion of the Σ5 STGB compared to the bulk is observed, while there is a mild HEDE effect on the Σ STGB. However, for a definite statement, the partitioning of H among these systems for a fixed chemical potential, and the solution energies at higher concentrations, especially at the Σ5 STGB, have to be determined.

To better understand the differences observed between the decohesion across bulk and GB planes, the Voronoi volume of the hydrogen atoms was calculated using the Voro++ code [45], at the highest concentration for each case. The volume of the hydrogen atoms at equilibrium in the tetrahedral space in the bulk is 6.3 Å^3^. In the GB structures, H has a volume of 6.6 Å^3^ in Σ3, and, in the more open Σ5, the Voronoi volume is 7.8 Å^3^. These results indicate a link between the available space around the H atom or free volume and its influence on the cohesive strength—structures with larger free volume around the H atom are less sensitive to H embrittlement. This is in agreement with the finding, that the reduction of the elastic constants of Fe in the presence of H is a volumetric effect [6]. This trend seems to be systematic, considering that the chosen structures differ significantly in the local geometric environment as well as their trapping energies of H. However, studies including other GB structures have shown, that not only the volume, but also the coordination polyhedra, influence the effect of H [2,11]. Thus, to develop a quantitative model more data is needed on the cohesive strength as a function of the local structure around the H atom.

**Figure 12:**
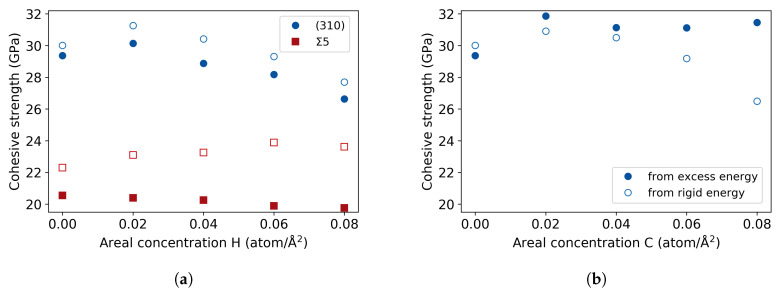
Theoretical cohesive strength vs. areal concentration from excess and rigid energies, for the (**a**) Σ5(310)[001] and (310) cleavage plane with H and (**b**) (310) plane with C.

Concerning the model aspects of the work, the cohesive zone model was successfully implemented for supercells with GB planes, taking into account the presence of two cohesive zones. An extra term to the excess length is added, which corresponds to the excess volume of the GB. In regard to the results from the different procedures of the *ab initio* tensile test, as proven by Van der Ven and Ceder [16], the cohesive strengths calculated from the rigid energies or the excess energy of the cohesive zone are equivalent for the pure single crystal. Here, it is shown that this extends to single crystal with H impurity atoms, as already assumed by Van der Ven and Ceder in the case of H in Al [15]. However, it is not the case for the GB structures where there are discrepancies between the results obtained with the two methods. To be able to judge to what extent this deviation is due to the excess volume of the GB, the cohesive strengths were also calculated at different H concentrations in the (310) cleavage plane (Figure 12a). Similarly to the (001) and (111) planes, the (310) results from the excess and relaxed energies provide the same trend with small numerical differences, whereas the trend at the Σ5(310) STGB seems to differ (for comparison, the data is repeated in Figure 12a)). Additionally, the hydrogen atoms were substituted with carbon to inspect the effect of the size of the impurity atoms (Figure 12b), resulting in different trends from rigid and relaxed procedures, with more significant numerical differences. These results indicate that the difference between rigid and relaxed procedures of the tensile test depend on the size of the distortions introduced by the defects, be it impurity atoms or an interface. Both, the rigid and relaxed, tensile tests approaches define two limits of a more realistic picture of separation, meaning that looking at both cases is necessary to form a better understanding of the decohesion phenomena.

## 5. Conclusions

Motivated by seemingly contradictory results in the literature, this study has investigated the extent of hydrogen enhanced decohesion in bcc Fe between adjacent (001) and (111) planes as well as at the Σ5(310)[001] and Σ3(112)[1$\bar{1}$0] STGBs. The cohesive zone model approach used in References [3,15] was followed using rigid grain shifts as well as rigid grain shifts followed by atomic relaxations. In case of the latter, the excess energy and length were determined, to avoid a dependency of the strength on the supercell size. The results obtained from tensile tests with and without the relaxation of the atomic positions lead to the same predictions of the effect of H on the cohesive strength of single crystal Fe, but they differ in the case of the strength of the Σ5 STGB. The discrepancies between both methods of tensile test are associated with the distortions introduced by the defects, indicating that in the case of GBs with a large excess volume, or bigger impurity atoms, the relaxation of the atomic positions can change the predicted trend in cohesion. Thus, we recommend the relaxation procedure to be applied when performing tensile tests for a system with defects, which introduce large and long ranging elastic distortions. At the same time, from a practical point of view, there is no extra effort to evaluate also the rigid grain shift results, which are the starting point of the relaxation procedure. In case that the deviation between both methods are small, the RGS results have the advantage, that they provide access to the complete energy vs. displacement curve.

Despite the discussed differences, both methods predict, that the single crystal cleavage planes are much more sensitive to an increase in H concentration than the two grain boundaries, especially than the Σ5(310)STGB, which is almost insensitive to the presence of H. This can be seen in both, the cohesive strength values and the work of separation. The effect of H on the cohesive strength of Fe can thus be attributed to the free volume around the H atom, depending on the coordination or density of atoms around H. Still, the investigation of a higher range of structures and of H concentration at the GB planes remains desirable to confirm these trends and to derive a quantitative model. In particular, at higher H concentration, the occupation of non-equivalent structural units and interstitial sites in the vicinity of the interface could lead to deviations from the linear trend. This requires the sampling of a large configurational space, which is the topic of a separate study.

## Figures and Tables

**Table 1:** Grain boundary energy and excess lengths of Σ5 and Σ3 STGB.

GB Structure	*γ_GB_* (J/m^2^)	Excess Length (Å)
Σ5(310)[001]	1.58	0.31
Σ3(112)[1$\bar{1}$0]	0.43	0.16

**Table 2:** Chemical and mechanical contributions to the change in cohesive energy.

Structure	ΔE^*cohesive*^ (J/m^2^)	ΔE_*chem*_ (J/m^2^)	ΔE_*mech*_ (J/m^2^)
(001)	−0.28	−0.19	−0.09
(111)	−0.22	−0.18	−0.04
Σ3(112)[1$\bar{1}$0]	−0.68	−0.57	−0.11
Σ5(310)[001]	−0.15	−0.16	0.01
